# Can *I* Choose a Throwable Object for *You*? Perceiving Affordances for Other Individuals

**DOI:** 10.3389/fpsyg.2019.02205

**Published:** 2019-09-27

**Authors:** Huichao Ji, Jing Samantha Pan

**Affiliations:** Department of Psychology, Sun Yat-sen University, Guangzhou, China

**Keywords:** affordance, throwing, actor-environment scaling, haptic perception, size-weight relation

## Abstract

Throwing is an important motor skill for human survival and societal development. It has been shown that throwers could select throwable balls for themselves and ball throwability was determined by its size and weight. In this study, we investigated whether throwers could perceive ball throwability for other throwers (experimental confederates) and whether the perceived throwability for others also followed a size-weight relation. Like other types of affordances, throwability entails a scaling between the thrower and the throwing object. This requires knowledge about the thrower and the object. In this study, knowledge about the objects was gained by hefting balls of various sizes and weights; knowledge about the throwers was gained by interacting with throwers in person (Experiment 1) and by viewing videos of confederates throwing (containing kinematic and anthropometric information) or photographs of the confederates standing (containing anthropometric information; Experiment 2). By comparing observers’ perceived throwability for others using various materials, we attempted to uncover whether scaling of throwability was based on kinematic or anthropometric information. In this study, participants ranked throwability of balls of various sizes and weights for confederates of different sexes and fitness levels. In all experimental conditions, observers’ ranking and confederates’ actual throwing performances yielded linear relationships with slopes close to 1 and moderate to high *r*^2^ values. These suggested that participants were able to accurately perceive throwability and choose throwable balls for the confederates. The throwable balls followed a size-weight relation, where bigger balls had to weigh more to be perceived as throwable as smaller balls. Furthermore, there was no difference between throwability perception based on in-person interaction, watching videos of confederates throwing and seeing pictures of the confederates standing. This suggested that the scaling of throwability was likely to be based on anthropometric information. These results enriched our understanding of whether one could perceive the action opportunities for others, and extended the canonical Gibsonian concept of affordance to a social setting and thus could be important for understanding team coordination in sports and interpersonal action collaboration in general.

## Introduction

Animals evolve to coordinate individual behaviors at a group level to increase the effectiveness of joint actions for better survival of the group (Herbert-Read, 2016). Similarly, humans help each other or coordinate with other members to achieve some common goal of the group. Underlying this kind of social interaction, an important assumption is that one perceives whether the other can accomplish an action or not. In other words, to initiate help or to collaborate, one has to perceive action possibilities of other individuals.

These action possibilities, known as affordances (Gibson, 1979, 1986; Turvey et al., 1981), are dispositional properties of objects, surfaces and events that are *functional, real*, *relational*, and *perceptible*. By “functional”, it means that affordances are the functional possibilities for actions (Warren and Whang, 1987). For example, a floor is flat and solid that a man may stand on it, then the floor has standing affordance or standability. Equivalently, standability is a functional property of the floor. By “real,” it means that affordances exist as facts and not subjective experiences; affordances are real properties in the environment and not relations between organisms and the environment. For example, salt is soluble in water regardless of whether water is present or not. Being soluble in water is a real property of salt; not a relation between salt and water. Similarly, a floor supports standing whether there is a man standing on it or not (Bingham, 2000). By “relational,” it means that affordances are determined by the fit between the environment and the organism’s action system, e.g., whether salt is soluble depends on what solvent is given and whether a floor is standable depends on who is trying to stand on it. By “perceptible,” it means that affordances are specified by optical information that is available in the medium of light for an observer to detect. One does not need to carry out the action to know the affordance.

The theory of affordance has received much empirical support using various types of actions and tasks. For example, Warren (1984) showed that when climbing stairs, the optimal stair height for climbing was about a quarter of the climber’s leg length, and the maximal stair height for climbing was about 88% of leg length. These fractions maintained regardless of climbers’ actual leg lengths. Moreover, observers were able to perceive optimal and maximal climbability for themselves, when looking at the stairs without actually climbing them. Therefore, climbability was relational and it was scaled between climbers’ bodies and environmental objects; climbability was perceptible without needing to perform the actions. Besides climbing, observers were also able to perceive whether apertures were passable (Warren and Whang, 1987), how throwable a ball was (Zhu and Bingham, 2008), how catchable a flying ball was (Postma et al., 2018), maximum height of reaching (Ramenzoni et al., 2005), maximum jump-and-reach heights (Pepping and Li, 2000), etc.

Zhu and Bingham (2008, 2010, 2011) studied throwing affordance. They created balls of six different sizes and within each size group, there were eight different weights. The participants threw the balls and ranked ball throwabilities after hefting in each size group. Their results showed that individuals who could throw long-distance (>30 m) were able to, after hefting, perceive ball throwability and pick out balls that they could throw to the farthest distances. Those who could not throw were unable to choose throwable balls, but after training with visual feedback (that is, participants saw the outcome of each throw during training) and once they became able to throw, they became able to perceive ball throwability. Thus, action capability and affordance perception were coupled. Furthermore, throwability was affected by both objects’ size and weight – a bigger object had to weigh more to be thrown as far as a smaller object and a bigger object had to weigh more to be perceived as throwable as a smaller object. Therefore, the size-weight relation was not only a cognitively impenetrable illusion (Charpentier, 1891), but also a functionally important piece of information that guides throwing. This information was detected via hefting.

Long-distance throwing is a unique human motor skill that greatly contributed to the survival of men and the forming of society during early ages, for example, throwing skills corresponded to social status and reproduction opportunities, and contributed a lot to the evolution of tool use, handedness, hunting, and complex language processing in early human societies (Knüsel, 1992; Hopkins et al., 1993; Young, 2003; Calvin, 2010). Zhu and Bingham’s series of studies supported that objects’ throwabilities are real, functional and perceptible for throwers and identified size-weight relation as the higher-order information for throwability. However, they have not shown if throwability is relational between individuals (or whether throwability is social), in other words, if one thrower can scale the size-weight relation and select throwable objects for another thrower.

To perceive affordances for others means to perceive the relations between other people and their environment. This is an important skill that people must have in order to socialize (Gibson, 1979, 1986, p. 141). Previous studies have suggested that in many actions, observers were able to perceive various types of affordances for others. For example, Wagman et al. (2018) studied perceiving others’ maximum reaching heights when experimental confederates stood on the floor, on the floor with a stepstool next to them, or on the floor with a stick next to them. Although actual reaching was never performed, observers were able to accurately perceive confederates’ reaching heights in all reaching conditions. This implied that affordances might be perceptible based on anthropometric information, i.e., body heights of confederates.

In another study of sitting affordance (Stoffregen et al., 1999), observers adjusted seat heights for tall or short experimental confederates. Participants were able to perceive maximum sittability and optimal sittability and set the seat higher in the maximum sittability condition. Participants also set the seat higher for the tall confederate, and set it lower for the short confederate. However, when dividing the adjusted seat heights by leg lengths, the ratios were different between short and tall confederates. This was contrary to findings (such as in Warren, 1984; Warren and Whang, 1987; Wagman et al., 2018) that suggested scaling between an actor and environment was based on some straightforward anthropometric dimensions, e.g., body height, leg length or shoulder widths. Furthermore, in this study (Stoffregen et al., 1999), participants were able to perceive sittability when interacting with the confederates in real time as well as when seeing point-light-displays of confederates’ movements onscreen. According to the authors, this suggested that information for affordance perception was extracted from movement kinematics.

Another type of well-studied affordance is reachability in the reach-with-jump (RWJ) task. Researchers have shown that observers accurately perceived reachability in an RWJ task for themselves as well as for others, and even when they had never seen the others jump (Ramenzoni V. et al., 2008; Ramenzoni V. C. et al., 2008; Weast et al., 2011). More importantly, when attaching weights to the actors and altering their jumping dynamics, observers were able to visually pick up this change from the actor’s movement kinematics and adjusted their reachability judgment in the RWJ trials accordingly (Ramenzoni V. et al., 2008). Follow-up studies showed that information that specified maximum reachability in RWJ was indeed extractable from walking kinematics, when walking was performed by a real person in real time (Ramenzoni V. C. et al., 2008) or displayed on screen with point lights (Weast-Knapp et al., 2019). Kinematic information allows the perception of affordance because it reflects the underlying dynamics of movement, and movement dynamics are determined by the physical properties of the actor and the environment. For example, a heavy man and a light man walking on the same hardwood floor will exhibit different kinematic patterns; a man walking on a sandy beach versus him walking on a hardwood floor will also exhibit different kinematic patterns. By detecting kinematic patterns, observers get to know the dynamics and the physical properties of the actor and the environment. Affordance entails actor-environment scaling, which is embedded in movement dynamics.

In the present study, we investigate whether observers can perceive objects’ throwability, via hefting, for other throwers and if so, with what information (anthropometric or kinematic) they perceive. Previous work has found that people can perceive throwabilities for themselves and can perceive some other types of affordance for other individuals, such as sitting affordance (Stoffregen et al., 1999) and RWJ affordance (Ramenzoni V. et al., 2008). We thus hypothesize that people can perceive throwabilities for other individuals. We modify and extend Zhu and Bingham’s (2008, 2010) studies to test whether observers are able to accurately rank ball throwability for four experimental confederates of different sexes and fitness levels. Participants heft and rank throwabilities of 16 balls (4 sizes × 4 weights) for each confederate. In Experiment 1, participants interact with the confederates in person and participants’ throwability ranking is compared with confederates’ own throwability ranking and with confederates’ actual throwing performance. In Experiment 2, participants gain information regarding confederates’ potential throwing capacity from different types of materials: full videos of confederates throwing, partial videos which only show the ballistic throwing actions but not the ball flight or landing positions, or photographs of confederates standing. Throwability ranking based on the different types of materials is compared with the confederates’ actual throwing performance. If observers perceive affordance based on action skills, then they should only be able to rank throwability when seeing the full videos of throwing. If observers perceive affordance from movement kinematics, they should be able to rank throwability from both full and partial videos (because both show the actions). If observers perceive throwability based on anthropometric information, then they should be able to rank throwability when seeing confederates’ full videos, partial videos or photographs, because all reveal confederates’ physique. Results from Experiment 1 inform whether throwability is scalable between individuals. Results from Experiment 2 inform whether throwability scaling is based on kinematic or anthropometric information.

## Experiment 1: Perceiving Affordances for Other People in Real Life

In this experiment, we investigated whether skilled throwers were able to perceive throwability for other throwers and if so, whether the perceived throwability was affected by the size-weight relation of throwing objects. We selected male and female experimental confederates of different fitness levels and asked participants to interact with them and hence rank throwability of balls of various sizes and weights for the confederates.

### Methods

#### Confederate Selection

Ten self-claimed skilled throwers volunteered to be the experimental confederates. All were right-handed. After signing the informed consent, their demographic information, anthropometric data, physical strength levels, and exercise routines were measured and recorded (see Appendix 1). They then came to a volleyball court and performed a series of throwing tasks. First, they threw a tennis ball (weight = 57 g, diameter = 6.7 cm) as a screening procedure. Only those who were able to throw (with an overhand throwing style) a tennis ball for 30 m or more in three consecutive trials qualified as skilled throwers in this experiment. All volunteers met this criterion and none was excluded. Next, the 10 volunteers threw a beanbag (size = 7 cm × 7 cm × 7 cm and weight = 65 g) and their throwing was recorded using an Apple iPad. Finally, we took full-body pictures of the volunteers standing up straight. Volunteers were able to take breaks in between trials. Each volunteer received ¥10 for participation in this phase. All experimental procedures in this study were approved by the Sun Yat-sen University Institutional Review Board.

Next, we sent the videos of the volunteers throwing beanbags and their full-body pictures to 65 adults (26 males), who were naïve to the purpose of the experiment and never returned for future testing, and asked them to rank the fitness level of the 10 volunteers on a scale from 1 (weakest) to 7 (strongest). We then selected the strongest male, the weakest male (fitness ranking scores differed significantly, *t* (64) = −16.912, *p* < 0.001), the strongest female, and the weakest female (fitness scores differed significantly, *t*(64) = 9.439, *p* < 0.001) to be involved in the following testing procedures. Each of the four selected confederates was paid ¥100 for their participation in the next experimental procedures.

#### Experimental Materials

The purpose of this experiment was to test the perception of throwability of balls of different sizes and weights. We inserted the lead into spherical Styrofoam shells and created 16 balls of four weights and four sizes. Stretchable white plastic tapes were used to wrap around the balls to produce the identical appearance and surface texture and to increase durability. A small mark (such as a tiny blue dot or a thin red line) was painted on each ball for the experimenter to differentiate and identify balls of the same size but different weights. This was to make data recording easier for the experimenter. During post-experimental debriefing, no participant reported having noticed the marks or considered them as meaningful in any means. We selected similar (but fewer) levels of weights and sizes as in Bingham et al. (1989), in order to make comparisons. Diameters of the balls were 6, 8, 10, and 12 cm. For each size, the smallest weights were 18, 45, 70, and 108 g, respectively. At each level of size, there were four different weights, and weight increment was by a factor of 1.55 (*W*_*n* + 1_ = *W*_*n*_ × 1.55). All balls were comfortably graspable. See Table 1.

**TABLE 1:** Sizes and weights of throwing objects.

**Diameters (cm)**			**Object weight (g)**	
6 cm	19	29	45	70
8 cm	45	70	108	168
10 cm	70	108	168	261
12 cm	108	168	259	402

#### Participants

Twenty right-handed experienced throwers (20 males, age range 18–23) participated in this experiment. All of them successfully threw a tennis ball, in the overhand style, for over 30 m in three consecutive trials. Participants had a normal or corrected-to-normal vision and had no known motor or visual impairment. They received ¥30 for participation. All participants were explained about the procedures, potential risks and benefits of the experiment and then they signed informed consent in accordance with the procedures approved by the Sun Yat-sen University Institutional Review Board.

#### Experimental Procedures

First, the four selected experimental confederates came to an open field. They did some stretch and warm-up before they threw the 16 balls of various sizes and weights in random order. The confederates were told to throw each ball as far as possible in an overarm style and took no more than one step before releasing the ball. They were allowed to rest in between throwing attempts. Confederates threw each of the 16 balls for three times and an experimenter measured and wrote down the throwing distance after each throw. Throwing distance was measured as that between the last step before releasing the ball and the first hit of the ball on the field. This process lasted for approximately half an hour for each confederate.

Next, the confederates entered separate rooms and interacted with experimental participants one at a time. Participants were told that their task was to rank balls according to how far they could be thrown by four individuals whom they were going to meet. While a participant and a confederate were in a private room, the participant was allowed to look at the confederate from all directions around him/her, read the physical fitness information collected for the confederate in the Physical Fitness Survey (Appendix 1), ask questions such as “how often do you work out?” or “do you lift weights?,” or even ask the confederate to throw a beanbag in the hallway outside of the room. However, the confederates were told not to answer questions directly related to the experiment, such as the ball weight or size she/he felt comfortable throwing. For curtesy reasons, participants were not allowed to touch the confederates. Participants took as much time as needed to gather information from and about the confederate before moving on to the next phase. Generally, it took a participant 5 min to complete this part. During the post-experiment debriefing, we asked and confirmed that the participants had not met or known the confederates before the experiment.

After interacting with the confederate, the participant began hefting and ranking four groups of 16 balls according to their throwability for each confederate. Each group consisted of four balls of the same size but different weights. The four groups were randomly presented to the participant, one group at a time. On each trial, a group of four balls with the same size but different weights were placed on a table in front of the participant. The participant hefted and then ranked the four balls of each size group according to the throwability, or how far the participant thought the balls could be thrown by the confederate. The hefting was done with the right arm (the throwing arm). When hefting, the participant held one of the four balls in the hand, kept the upper arm next to their torso, and rotated the elbow and the wrist up and down, while keeping the eyes open. The participant was allowed to begin with any ball, heft in any order, and revisit previously hefted balls. The hefting process was not timed and the participant took as much time as needed. The experimenter was present during this process to instruct, demonstrate and correct the hefting movement, if needed. After hefting, the participant ranked the throwability of the four balls by placing them in a long box (size = 80 cm × 20 cm × 20 cm), which was divided into four equally sized slots and labeled “the most throwable,” “the second most throwable,” “the third most throwable,” and “the least throwable.” The experimenter hence noted down the throwability ranking. The same hefting and judgment procedures were repeated for all four ball size groups for all four confederates. Participants were allowed to take breaks when needed.

Finally, after all the participants’ data were collected in the experiment, the four confederates came back to the lab and hefted and ranked the balls according to their throwability. This was done in the exact same way as how participants ranked ball throwability. There was approximately a month gap between when confederates threw the balls and when they ranked the ball throwability.

#### Data Analysis

We first examined confederates’ throwing and self-ranking performance, using ANOVA with their throwing distance as the dependent variable and sex and fitness level as the independent variables. Next, we compared confederates’ throwing to participants’ ranking to assess throwability perceived by others. We converted participants’ ranking into a composite score, the mean weight average, which was a weighted average of the three most throwable balls. We did regression between sizes and weights of the most throwable balls to examine the effect of size-weight relation. We correlated participants’ ranking and actual throwing to study the accuracy of participants’ perception.

### Results and Discussion

The purposes of this experiment were to test, first, if observers, who were experienced throwers, were able to heft and select throwable balls for other throwers after interacting with them in real life; and if the perceived throwability for others followed a size-weight relation as reported by Bingham and colleagues (Bingham et al., 1989; Zhu and Bingham, 2008).

#### Confederates’ Throwing and Self-Ranking Performance

**FIGURE 1:**
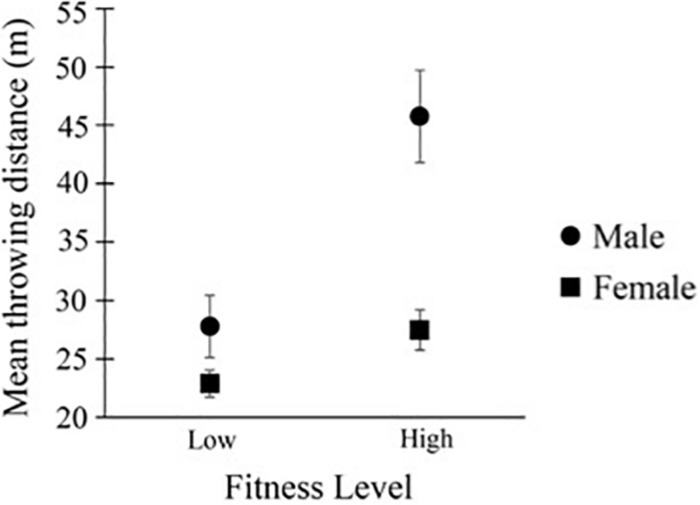
Throwing performance of the four selected confederates. On the *y*-axis was the mean throwing distances of the most throwable balls of all size groups. Error bars = 1 SE.

We first looked at the four selected confederates’ throwing performance and found that when throwing the testing objects, the distances of their most throwable balls in each size group were affected by sex [*F*(1, 44) = 181.10, *p* < 0.001; Mean distance_*Male*_ = 36.78 m, SD_*Male*_ = 0.61; Mean distance_*Female*_ = 25.18 m, SD_*Female*_ = 0.61], and fitness level [*F*(1, 44) = 171.60, *p* < 0.001; Mean distance_*Low Fitness*_ = 25.33 m, SD_*Low Fitness*_ = 0.61; Mean distance_*High Fitness*_ = 36.62 m, SD_*High Fitness*_ = 0.61], and sex X fitness interaction [*F*(1, 44) = 59.37, *p* < 0.001]. See Figure 1. Note that the division of confederates into high versus low fitness levels was judged by naïve observers who saw the pictures and the videos of the confederates throwing beanbags. This result showed that there was indeed an actual difference in throwing performance using the testing objects for the rated-as-fit and rated-as-less-fit confederates.

**FIGURE 2:**
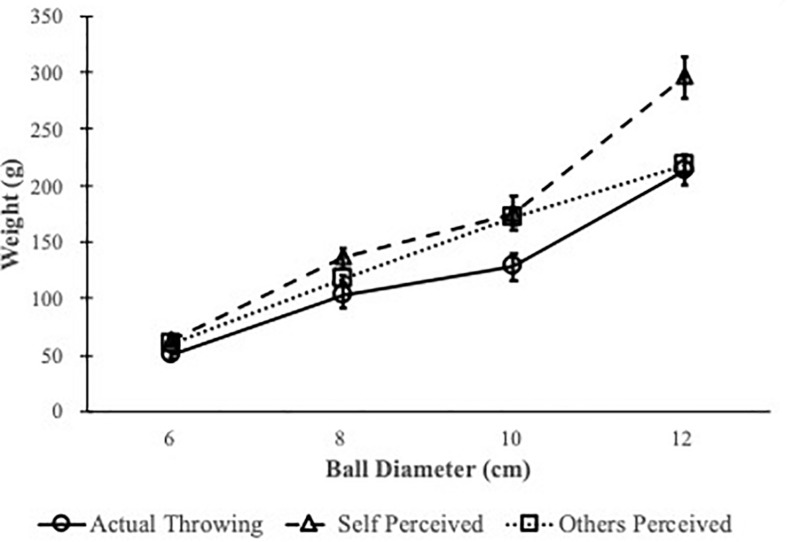
Sizes and weights of the most throwable balls for the confederates, as thrown by themselves (circle), as ranked by themselves (triangle), and as ranked by the participants (square). Error bar = 1 SE.

The confederates also ranked the four balls of different weights in each size group for themselves according to ball throwability. The confederates’ perceived throwability coincided with their actual throwing performance and both exhibited the size-weight relation. See Figure 2. Looking at the confederates’ best throwing trials, as the ball size increased, the farthest-thrown balls weighted increasingly more. Congruently, after hefting, confederates selected heavier balls to be the most throwable for themselves, as ball size increased. There was no slope or intercept difference when fitting least-squared lines to the throwing and perceived data (slope_*throw*_ = 25.75, slope_*self*p*erceived*_ = 36.87, *p*_*slope diff*_ = 0.14; intercept_*throw*_ = −108.03, intercept_*self*__p__*erceived*_ = −164.34, *p*_*int diff*_ = 0.42). Additionally, the correlation between perceived throwability and actual throwing distance rank was significant (Spearman ρ = 0.663, *p* < 0.001). This replicated previous results that those who were able to throw were able to perceive objects’ throwability for themselves and throwability was affected by size-weight relation.

#### Participants’ Ranking Performance

We first selected the balls that were ranked as the most throwable for the confederates by the participants and compare them with balls that were in fact thrown for the farthest distance (Figure 2). When fitting least-squared lines to the weights and sizes of the most throwable balls, there was no slope or intercept difference between the confederates’ actual throwing performance and participants’ selection (slope_*throw*_ = 25.75, slope_*o*__*thers*__p__*erceived*_ = 26.35, *p*_*slope diff*_ = 0.80; intercept_*throw*_ = −108.03, intercept_*others*p*erceived*_ = −95.5, *p*_*int diff*_ = 0.91).

Next, looking at the ranking of all four balls of each size group, participants’ ranking was correlated with the confederates’ throwing performance (Spearman ρ = 0.581, *p* < 0.001). Bingham and colleagues (Bingham et al., 1989; Zhu and Bingham, 2008) combined the top three choices of ball weights by multiplying different coefficients to get a mean preference of weight for each ball size:

(1) $$\mathrm{Mean}\,\mathrm{weight}\,\mathrm{preference}=0.5\times w_{1}+0.33\times w_{2}+0.16\times w_{3}$$

where *w*_1_ is the weight of the most throwable ball, *w*_2_ is the weight of the second most throwable ball and *w*_3_ is the weight of the third most throwable ball.

**FIGURE 3:**
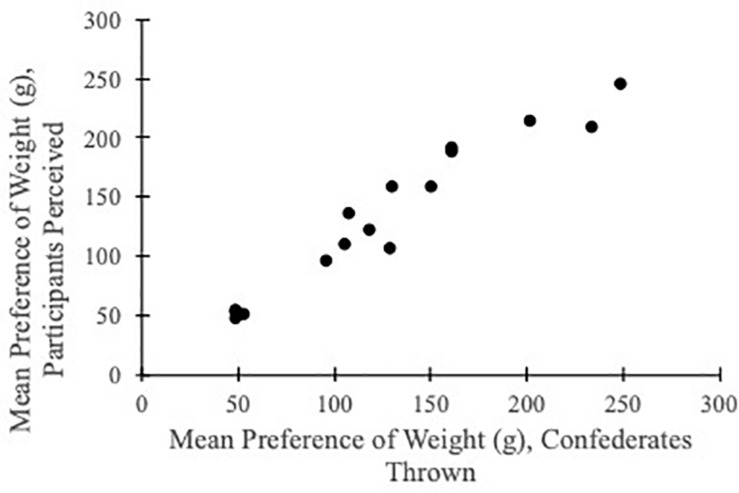
Mean weight preference scores of confederates actual throwing performance and participants’ perceived throwability.

**FIGURE 4:**
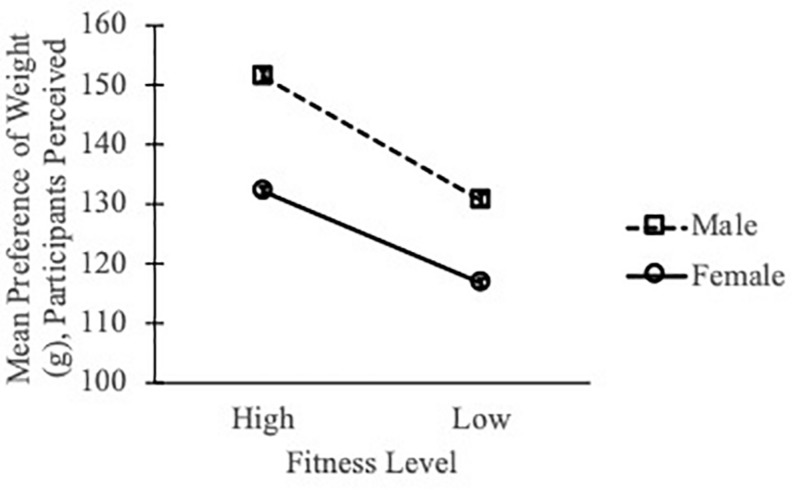
The perceived mean weight preference was differentiated between fitness levels and sexes. Error bars = 1 SE.

Following Eq. 1, we computed the mean weight preference scores for participants’ selection of ball weights and for confederates’ actual throwing performance. Both were normally distributed and they were significantly correlated (Pearson’s *r* = 0.966, *p* < 0.001, Figure 3). A linear relationship between participants’ perceived mean weight of preference and confederates’ performance had a slope of 0.98 (not significantly different from 1) and intercept of 6.95 (not significantly different from 0). Furthermore, the perceived mean preference was different between confederates of different sexes [*F*(1, 19) = 19.83, *p* < 0.001, *η_*p*_*^2^ = 0.070] and fitness levels [*F*(1, 19) = 23.84, *p* < 0.001, *η_*p*_*^2^ = 0.082]. There was no sex X fitness level interaction effect (*p* = 0.45). See Figure 4. These results suggested that observers accurately ranked ball throwability for other throwers and participants were able to scale throwability according to confederates’ sexes and fitness levels.

Altogether, results from this experiment suggested that when participants hefted and ranked ball throwability for the confederates, when confederates hefted and ranked the balls for themselves, and when the confederates actually threw the balls, the most throwable balls always followed a size-weight relationship. Bigger balls had to weigh more to be the most throwable in all cases. Participants were able to perceive, via hefting, ball throwabilities for the confederates and their ranking reflected sex and fitness level distinctions of the confederates.

## Experiment 2: Perceiving Affordances for Other People Based on Indirect Information

In Experiment 2, we investigated whether observers could perceive throwability for other individuals based on information acquired indirectly from videos or photographs. Specifically, observers came to know the throwers from various types of materials and ranked ball throwability for them. The types of materials contained different information: full-body photographs contained anthropometric information of confederates’ physique, partial videos of confederates throwing (which were cut off after the ball left the thrower’s hand) contained movement kinematics in addition to anthropometrics, and full videos of confederates’ throwing reflected throwing skills in addition to kinematics and anthropometrics. Comparing affordance ranking with these materials would reveal what kind of information guides the perception of throwability.

### Methods

#### Participants

Twenty-four right-handed students from Sun Yat-sen University were recruited (21 males, age range 18–23). All participants reported having no motor or visual impairment. We screened the participants by asking them to throw a tennis ball for three times. All were able to throw >30 m in three consecutive throwing attempts and were included in the experiment. Participants were explained about the procedures, potential risks and benefits of the experiment and then they signed informed consent. Participants received ¥30 after the experiment as compensation for their time and effort.

#### Confederates

The four selected confederates from Experiment 1 were involved in this experiment. Their throwing videos and photographs were used by the participants to rank ball throwabilities for them. Specifically, participants had access to three types of materials that gave information regarding the confederates’ throwing-related capabilities: first, full videos of confederates throwing beanbags (size = 7 cm^3^; weight = 65 g), which were recorded during the confederate selection phase of Experiment 1; second, partial videos of beanbag throwing, which were edited from the full videos revealing only the throwing action but not the throwing outcomes (i.e., ball flight and landing distance); third, full-body pictures of the confederates, which portrayed confederates standing but not throwing.

#### Experimental Materials

Balls (4 weights × 4 sizes) that were used in Experiment 1 were used again in this experiment.

#### Procedures

Participants randomly received one type of materials to gain knowledge of the confederates. They were instructed to rank throwability of balls of different weights and sizes in the same way as in Experiment 1. Participants hefted and ranked 16 balls for each of the four confederates, using one type of materials given to them.

In this experiment, each participant received 1 type of materials regarding the confederates throwing capabilities. A participant had to rank four balls in each size group, for four size groups and for four confederates (of 2 sexes and 2 levels of fitness). Each participant completed 64 trials of hefting and ranking. “Type of material” (full video, partial video and photograph) was a between-subject variable. The 24 participants were divided evenly and randomly into three groups receiving different types of materials.

#### Data Analysis

We first compared confederates’ throwing to participants’ ranking to assess throwability perceived by others in different materials, using correlation between participants’ ranking and actual throwing distance rank. We then performed an omnibus ANOVA on the mean weight average to study the perceived mean weight preference, with the type of material as a between-subject variable, and confederates’ sex and fitness level and ball size as repeated factors.

### Results and Discussion

**FIGURE 5:**
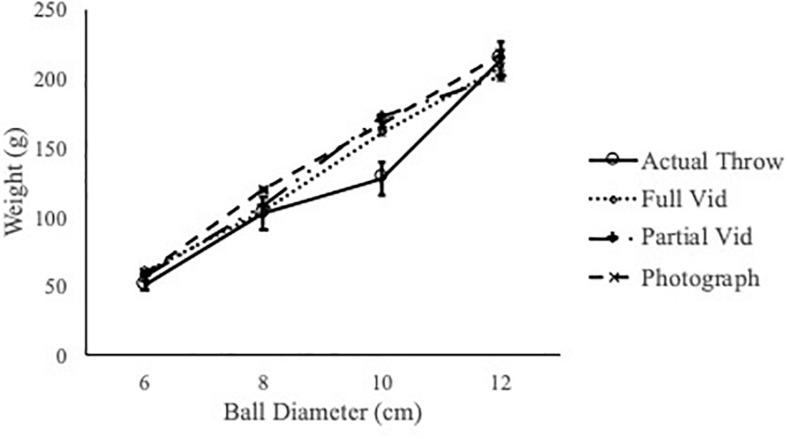
Participants selected the mast throwable ball in each size group, after viewing different types of materials that depicted the confederates. The selected-as-most-throwable balls followed a size-weight relation that was the same as confederates’ actual throwing performance. Error bar = 1 SE.

First, when participants saw full videos of confederates throwing, partial videos of confederates throwing and photographs of confederates standing, they were all able to select the most throwable balls for them. The most throwable balls selected by the participants showed a size-weight relation that was the same as confederates’ actual throwing performance (Figure 5).

Looking at the ranking of all four balls of each size group, with all types of materials, participants’ ranking was correlated with the confederates’ throwing performance. See Table 2 for a summary of the rank order correlations.

**TABLE 2:** Correlations between ball throwability ranking with each type of materials and actual throwing performance.

	**Spearman’s rho**	***p***
**Experiment 1**		
Real Person	0.581	<0.001
**Experiment 2**		
Full video	0.542	<0.001
Partial Video	0.491	<0.001
Photograph	0.559	<0.001
		

We then calculated the mean weight preference scores using Eq. 1 and performed an omnibus ANOVA, with the type of material as a between-subject variable, and confederates’ sex and fitness level and ball size as repeated factors. Results showed that mean weight preference was affected by confederates’ fitness level [*F*(1, 21) = 33.54, *p* < 0.001,*η_*p*_*^2^ = 0.071] and sex [*F*(1, 21) = 39.11, *p* < 0.001, *η_*p*_*^2^ = 0.095] and ball size [*F*(3, 63) = 295.65, *p* < 0.001, *η_*p*_*^2^ = 0.78]. The pairwise two-way interactions between these three factors were also significant (*p* < 0.05 in all cases), but the effect sizes were small (*η_*p*_*^2^ < 0.04 in all cases). There was no significant three-way interaction. Moreover, the type of material was not a significant factor (*p* = 0.98), which implied that the perceived mean weight preference was not different when judging from full videos, partial videos or photographs. Combining these results with Experiment 1’s, there was no difference between perceived mean weight preference when interacting with the confederates in person versus ranking based on videos or photographs [*F*(3, 40) = 0.25, *p* = 0.86]. Comparing perceived mean weight preference with each type of materials to the actual mean weight preference (calculated based on confederates’ throwing data using Equation 1), the fitted least-squared lines had similar slopes and intercepts (Table 3), which suggested that when interacting with real confederates or when viewing their full videos, partial videos or pictures, participants were always able to perceive throwability.

**TABLE 3:** Mean weight preference scores based on participants’ ranking regressed to mean weight preference scores of actual throwing performance.

	**Slope**	**Intercept**	***r*^2^**
Real person	0.98	6.95	0.94
Full video	0.93	7.43	0.91
Partial video	0.92	9.33	0.92
Photographs	1	0.9	0.95

*Pairwise, there was no difference between the slopes and intercepts.*

## General Discussion

In the present study, we conducted two experiments to investigate whether observers could perceive objects’ throwability, via hefting, for other throwers and if so, what information (anthropometric or kinematic) they would use in order to perceive. Our results showed that those who could throw could heft to select throwable objects for other throwers and bigger balls had to weigh more to be perceived as throwable as smaller balls.

Being able to perceive affordances for the self is critical for safely and successfully performing actions; being able to perceive affordances for others is a prerequisite for collaborative and coordinated group behaviors. In the task of long-distance throwing, it has been shown that throwers were able to perceive throwability and select the appropriate objects for the self via hefting (Zhu and Bingham, 2008, 2010). In this study, we further established that observers were able to perceive throwability for others. Consistent with Zhu and Bingham’s results, throwability perceived by others was also determined by a size-weight relation, where bigger objects had to weigh more to be perceived as equally throwable as smaller objects. Furthermore, when interacting in person, watching full throwing videos, watching partial throwing videos or seeing photographs of the confederates, participants were always able to perceive and rank ball throwability. This suggested that information on action skills or movement kinematics was not necessary and anthropometric information alone might be sufficient for observers to perceive throwability for the confederates.

We first showed that the selected confederates were indeed different in their throwing behaviors, where the rated-as-fit confederates threw farther than the rated-as-less fit confederates and male confederates threw farther than the females. These differences allowed us to analyze and compare the choice of confederates as well as participants. So the actual throwing performance was used as the standard for subsequent comparisons.

When analyzing participants’ throwability ranking, we examined the most throwable balls, the rank order of all four balls in each size group and the mean weight preference scores, which was a weighted average of the top three selections. The most throwable balls selected for the confederates upon interacting in person, watching full or partial videos or seeing full-body pictures were all coincided with confederates’ actual throwing performance (Figures 2, 5) and bigger balls weighed more to be perceived as throwable as smaller balls. Therefore, observers were sensitive to the size-weight relation and used it when ranking throwability for others.

Throwability is a property of the throwing target. In this study, how far a ball can be thrown and how throwable a ball is perceived to be are determined by ball size and ball weight, because the size and weight affect the throwing dynamics. Observers are able to perceive throwability by hefting because hefting and throwing encompass similar movement dynamics of the body (Zhu and Bingham, 2010). In other words, ball size and ball weight form one single higher-order variable that affects hefting and throwing dynamics. That is possibly why only those who can throw can perceive throwability via hefting. That is also why we only include participants who can throw long distance in these experiments. Skilled throwers directly perceive the size-weight relation as a higher-order information variable via a smart perceptual mechanism (Runeson, 1977). The smart perceptual mechanism is evolved to detect higher-order information directly, without processing its constituent parts, and apply it for guiding actions. For example, Thomas et al. (2018) showed that the perceived reachability in a RWJ task was independent of the perceived jumpability in a jumping task or reachablity in a reaching task. Affordance in RWJ is not a linear combination of reaching and jumping affordances. In the same vein, when perceiving throwability for other throwers, the size-weight relation should be scaled as one variable, instead of scaling the size first and then taking into account the weight or vice versa. Perceiving and scaling size or weight individually are not essential for perceiving throwability for others.

In this study, participants were not only able to select the most throwable ball, but also able to rank all balls’ throwability. This was reflected in the significant correlations between participants’ perceived throwability rank orders and confederates’ actual throwing rank orders, which were found in all conditions with in-person interaction, full or partial videos or full-body pictures (Table 2).

Considering the three most throwable balls, the mean weight preference scores between others’ perception and actual throwing were highly consistent with linear trends of slopes close to 1 (Figure 3 and Table 3). Given that there were only four balls in each size group, this means that perceived throwability for all balls were highly compatible with confederates’ actual action performance. Importantly, in both experiments, the mean weight preference scores were differentiated between confederates’ sexes and fitness levels. This means that participants were sensitive to scale perceived affordance according to the differences between the confederates and participants were indeed ranking throwability for them. Furthermore, there were altogether 44 participants (of different sex, fitness level and body size) in the two experiments, and all participants ranked ball throwability in a consistent way that was abiding by the confederates’ throwing performance. The high correlation between participants within each experimental condition (Table 3, “*r*^2^” column), the high similarity between confederates’ throwing and participants’ perception (Table 3, “slope” column) and the reliable distinctions between perceived throwability for males versus females [*F*(1, 40) = 53.2, *p* < 0.001] and for fit versus less-fit confederates [*F*(1, 40) = 47.5, *p* < 0.001] convergently suggest that participants in these experiments were perceiving and ranking throwability for the four confederates (instead of for themselves). Throwability is relational and it requires scaling between a throwing object and the thrower. In early studies of affordances, Warren showed that the scaling was based on anthropometric information, for example, the optimal or critical climbable stair height was scaled to a climber’s leg length (Warren, 1984) and the optimal or critical passable aperture width was scaled to a walker’s shoulder width (Warren and Whang, 1987). However, more recent studies suggested that when perceiving affordances for other individuals, actor-based scaling (i.e., scaling based on body dimensions) did not hold (Stoffregen et al., 1999) and instead scaling was action-based or based on kinematic information (Weast et al., 2014; Weast-Knapp et al., 2019).

Results from Experiment 2 supported the actor-based scaling hypothesis. The result that relying on static images, which provided only anthropometric information but not action-related kinematics, participants were able to accurately perceive throwability, suggested that anthropometric information was sufficient, at least for this task. Adding kinematic information by presenting videos of throwing did not further improve the perception. Of course, this might be because the experimental task was relatively easy and there was a ceiling effect that rendered additional kinematic information unnecessary. In complex affordance perception tasks, kinematic information could still be useful.

In this study, we showed that observers were able to rank ball throwability for other throwers. However, *how* they rank is a question that requires further exploration. Specifically, observers perceive throwability for others based on perceptual information or via cognitive scaling is uncertain. Zhu and Bingham (2008) claimed that the perception of throwability is based on felt heaviness, which is a kind of haptically detected information for perception. Among balls of the same size, a thrower detects his or her most preferred felt heaviness. Felt heaviness as perceptual information is then generalized to balls of other sizes following a size-weight relation. Central to their claim, the perceptual information is scalable and the perceptual process is direct. Because information for throwability was scalable and generalizable across objects, it could also be scaled and generalized for different throwers. Thus, observers in the current study could have haptically detected felt heaviness of balls and scaled it to map to the confederates’ anthropometrics. This is a direct process that involved scaling of information and the perception of throwability for the self was not required. An alternative hypothesis of how the affordance scaling might be done was via cognitive scaling that is mediated by perceiving affordance for the self. When throwers (such as the participants in this study) rank throwability for other throwers (such as the confederates in this study), the participants could have first perceived ball throwability for themselves and then scaled it up or down for the confederates after looking at and comparing between the confederates and themselves on some dimensions such as body size, strength levels or throwing skills. In other words, ranking ball throwability for others relied on a combination of the participants’ haptic perception, motor experience and visually perceived features of the confederates. Observers did not directly perceive throwability for others, but took an indirect, two-step route. The premise of this approach is that one has to perceive throwability for the self before perceiving throwability for others. The first hypothesis (Zhu and Bingham’s original claim) is rooted in the Gibsonian tenet of information-based direction perception; the second hypothesis is cognitive in nature. Indeed, *how* observers may perceive affordance for others is a question that manifests important meta-theoretical discourse and demands further investigation. Future studies should specifically compare the perception of throwability for others and that for the selves to rule out one of the two hypotheses.

## Conclusion

The current study extended Zhu and Bingham’s (2008, 2010) studies and showed that those who could throw could heft to select throwable objects for themselves as well as for other throwers. Object throwability was determined both by its size and its weight. The size-weight relation was functional in choosing throwable objects for others. Furthermore, results from the current study suggested that perceived throwability was scaled to throwers according to their throwing capabilities. The scaling was based on anthropometric information that was detectable from the physical appearance of the throwers.

## Data Availability Statement

The datasets generated for this study are available on request to the corresponding author.

## Ethics Statement

The studies involving human participants were reviewed and approved by Institutional Review Board, Department of Psychology, Sun Yat-sen University. The patients/participants provided their written informed consent to participate in this study.

## Author Contributions

HJ conducted the experiments, analyzed the data, and wrote the first draft of the manuscript. JP designed the experiments, analyzed the data, and edited the manuscript.

## Conflict of Interest

The authors declare that the research was conducted in the absence of any commercial or financial relationships that could be construed as a potential conflict of interest.

## Appendix 1: Physical Fitness Survey

1. Participant number ______.

2. Age ______.

3. Sex ______.

4. Height: ______cm.

5. Weight: ______kg.

6. Upper arm circumference: ______ cm.

7. Arm length: ______ cm.

8. Have you had any upper body training (such as baseball, basketball, badminton, weight lifting etc.) in the past 6 months? ______.

9. How often do you participate in the above training? ______. (never/occasionally/often/almost every day)

10. What is the maximum weight that you can lift with one hand? ______kg.
